# Probing cellular health at the muscle level—Multi‐frequency bioimpedance in Parkinson's disease

**DOI:** 10.14814/phy2.15465

**Published:** 2022-10-05

**Authors:** Marko Celicanin, Adrian Paul Harrison, Jack Kvistgaard Olsen, Lise Korbo, Annemette Løkkegård, Charlotte Bjerg Petersen, Bente Danneskiold‐Samsøe, Hartwig Roman Siebner, Tihomir Ilic, Else Marie Bartels

**Affiliations:** ^1^ Department of Neurology Copenhagen University Hospital, Bispebjerg and Frederiksberg Copenhagen Denmark; ^2^ The Parker Institute, Copenhagen University Hospital, Bispebjerg and Frederiksberg Copenhagen Denmark; ^3^ Faculty of Health and Medical Sciences University of Copenhagen, IVH, PAS (Excitable Tissues & Biomechanics) Copenhagen Denmark; ^4^ Danish Research Centre for Magnetic Resonance, Centre for Functional and Diagnostic Imaging and Research Copenhagen University Hospital Amager and Hvidovre Hvidovre Denmark; ^5^ Department of Neurophysiology, Medical Faculty of Military Medical Academy University of Defense Belgrade Serbia

**Keywords:** bioimpedance, multi‐frequency, muscle tonus, muscles, Parkinson's disease

## Abstract

Bioimpedance (mfBIA) non‐invasively assesses cellular muscle health. Our aim was to explore whether mfBIA captures abnormal cellular muscle health in patients with Parkinson's Disease (PD) and how such changes are modulated with the use of Parkinson's medication. In patients with PD (*n* = 20) mfBIA measurements were made of biceps brachii, triceps, and extensor carpi radialis longus muscles of the more affected arm whilst at rest, using a mobile mfBIA device (IMPEDIMED, Australia). mfBIA and assessment of motor symptoms were performed in a pragmatic off‐medication state, as well as one and 3 h after oral intake of 200 mg levodopa. Age and sex‐matched healthy subjects (HC; *n* = 20) served as controls. PD and HC mfBIA parameters were compared by applying an unpaired two‐tailed adjusted *t*‐test and ANOVA with Tukey's correction for multiple comparisons (*p* ≤ 0.05). The PD group consisted of 13 men (71 ± 17 years) and 7 women (65 ± 7 years). Independent of medication, internal (R_i_) and external resistance (R_e_) were found to be significantly higher, and membrane capacitance (Mc) significantly lower, in *m.biceps brachii* in PD subjects compared to HC. Center frequency (fc) was significantly higher in *m.biceps brachii* of PD subjects in the “medication‐off” state. There was no difference between PD and HC in mfBIA parameters in the measured extensor muscles. The upper limb flexor muscle shows a difference in mfBIA parameters in PD compared to HC. mfBIA may be useful in the diagnosis and assessment of PD patients and is objective, non‐invasive, reliable, and easy to use.

## Abstract 2

Bioimpedance (mfBIA) is an objective, non‐invasive, reliable method when assessing the condition of upper limb muscles in Parkinson's patients. For M. Biceps brachii in PD subjects, internal (Ri) and external resistance (Re) were found to be significantly higher, and membrane capacitance (Mc) significantly lower, compared to healthy subjects. Centre frequency (fc) is higher in PD prior to PD medication, but decreases significantly following medication.

## 1 INTRODUCTION

Parkinson's disease (PD) is a progressive neurodegenerative disease, clinically characterized by motor and non‐motor symptoms. The cardinal motor symptoms are tremors, bradykinesia, rigidity, and postural instability. The main non‐motor symptoms are cognitive changes, as well as constipation and fatigue (Postuma et al., 2015; Russo et al., 2022). Moreover, patients with Parkinson's disease often experience a lowering of muscle power, weakness, and fatigability, beyond the physiological age‐related changes (Allen et al., 2009; Cano‐de‐la‐Cuerda et al., 2010; Herlofson & Kluger, 2017; Siciliano et al., 2018; Sijobert et al., 2017).

The main pathophysiology of PD is recognized as being associated with dopamine depletion in the basal ganglia, inducing disturbances in the connections to other parts of the brain, such as the thalamus and the motor cortex (Obeso et al., 2008). Muscle pathophysiology in PD is related to this impaired brain function, but some evidence also suggests the involvement of the peripheral nervous system (Comi et al., 2014). Patients with PD are found to have an altered muscle tone in both flexor and extensor muscle groups (Fung et al., 2000). Furthermore, studies with electromyography have demonstrated a low threshold for involuntary muscle contractions, which are present even when the muscle is relaxed (Cantello, 1995; Fung et al., 2000).

A deeper understanding of general muscle health in PD may help design a more focused treatment for the individual patient. Multi‐frequency bioimpedance (mf‐BIA) is a non‐invasive and quick method to assess muscle condition (Bartels et al., 2015; Bartels et al., 2019; Harrison et al., 2015). mf‐BIA has been used recently to assess muscle health as well as cellular metabolism (Bartels et al., 2019). It may therefore be a good diagnostic tool for regular assessment of PD patients, both in the clinic and during home visits.

The aim of this study was therefore to characterize muscle condition in patients with PD compared to healthy subjects by means of bioimpedance. Since PD muscles are known to be more rigid at rest compared with healthy muscles, we would expect higher mfBIA center frequency (fc) parameters and extracellular resistance (Re). One may also anticipate changes in cell metabolism as a result of the increased rigidity. Hence, this study has tested the following hypotheses; (1) Cellular energy metabolism is higher in PD muscle, as a result of increased rigidity, compared with healthy controls, such changes also being commensurate with membrane changes, measured as a higher intracellular resistance (Ri) and a lower membrane capacitance (Mc), and (2) Increased muscle rigidity in PD subjects manifests itself by both higher fc and Re values when compared with healthy controls.

## 2 MATERIALS AND METHODS

The bioimpedance study was part of a larger study, that also involved acoustic myography measurements, and the acoustic myography data (manuscript submitted).

### 2.1 Subjects

#### 2.1.1 Patients

Twenty patients with PD according to UK Parkinson's disease Society Brain Bank Criteria (Berardelli et al., 2013) were recruited at the Department of Neurology, Copenhagen University Hospital, Bispebjerg and Frederiksberg, Denmark.

##### Inclusion criteria for patients

- Parkinson's disease
- The Montreal Cognitive assessment (MoCa) >25 (Rossetti et al., 2011)
- Duration of disease <10 years
- Able to understand instructions in Danish and to answer questionnaires in Danish
- Mono/multi‐treated with PD drugs (Dopamine replacement drugs)
- Being able to be free of medicine for 12 h
- Pain reported in the normal range and pattern for healthy subjects from answering the PainDETECT Questionnaire (PD‐Q) ©2005Pfizer Pharma GmbH (Freynhagen et al., 2006) prior to participation

##### Exclusion criteria for patients

- PD drug‐induced dyskinesia (involuntary movements)
- Deep brain stimulation (DBS)

#### 2.1.2 Healthy controls

Twenty age and sex‐matched healthy subjects were recruited by advertising via the hospital's homepage. Prior to the enrolment, the control subjects were screened via a telephone interview to make sure they fulfilled the inclusion criteria.

##### Inclusion criteria for healthy controls

- 18.5 < BMI < 30
- Being healthy according to an examination by a physician
- Reporting of no chronic neuromuscular or movement disorder, no earlier history of affection of neuro system, or no present illness
- Pain reported in the normal range and pattern for healthy subjects from answering the PainDETECT Questionnaire (PD‐Q) ©2005Pfizer Pharma GmbH (Freynhagen et al., 2006) prior to participation
- Good understanding of the Danish language

### 2.2 Measurement schedule

The recruitment of patients occurred during consultations in the hospital's outpatient clinic. All aspects of the study were explained, and all recruited patients had the possibility to ask further questions. All patients had a minimum of one day to consider their participation in the study. After they decided to participate in the study by email or phone call, they were visited at home the day before measurements were performed. There they signed the informed consent prior to further assessment and were clinically assessed using the Unified Parkinson's Disease Rating Scale (UPDRS) (Perlmutter, 2009), including the Hoehn & Yahr rating (Goetz et al., 2004), MoCa test (Rossetti et al., 2011) and PainDetect questionnaire (Freynhagen et al., 2006).

The MoCa is a cognitive test applied to exclude patients who were not able to understand the study procedures and were not capable of giving informed consent to participate. The PainDetect is a questionnaire used to test that pain is not in a range that would affect muscle use. All participants were tested with this prior to the study start.

The following day, the patient's mf‐BIA parameters were measured at the outpatient clinic of the Department for Neurology at Copenhagen University Bispebjerg Hospital. The first measurement was performed in “pragmatic off‐medication”, the state achieved after 12 h period of fasting and not taking Parkinson's medication, corresponding to three times the half‐life for the use drug levodopa. The motor assessments by the UPDRS rating scale were performed prior to every set of mf‐BIA measurements. After the first mf‐BIA measurement in “pragmatic OFF” all patient subjects took an oral 200 mg dose of Levodopa and ate a breakfast half an hour after oral drug intake. The second and third mf‐BIA measurements were carried out precisely 1 and 3 h after levodopa intake and followed the second and third UPDRS motor assessments, respectively.

For the control subjects, measurements were carried out at one visit. Prior to their visit, the control subjects gave their informed consent and filled out the PainDetect questionnaire. Prior to muscle measurements, the subjects were assessed using UPDRS and subsequently bioimpedance measurements were carried out on m.biceps, m.triceps, and m.extensor carpi radialis longus. An attempt was made to also measure from m.abductor pollicis brevis, but due to its small size this proved inconsistent and data are not included as a result.

### 2.3 Multi‐frequency bioimpedance (mfBIA)

The selected muscles were assessed for mf‐BIA using an ImpediMed Inc tetra‐polar bioimpedance spectroscopy unit (Impedimed SFB7) and matching ImpediMed electrodes. mf‐BIA measurements were carried out with the subject in a sitting position, and it was ensured that the subject was kept free of all metal surfaces. A precisely determined anatomical area was localized for measurements on each of the muscles. In every case, the four electrodes were placed onto sites at the end of the muscle body, two per end, according to the manufacturer's recommendation. For all electrode placements, the outer two electrodes provided the electrical field, and the inner pair served as sensing electrodes measuring the difference between the two sites (mV). Measurements were carried out at a frequency range of 4–1000 kHz with a 6× repeat.

### 2.4 Measuring sequence

#### 2.4.1 Patients

The first set of measurements was carried out when the patient arrived at the clinic (08:00–09:00 am) in a fasted state and unmedicated for the previous 12 h. Following the mf‐BIA measurement, medicine was given, and then half an hour later the patient received breakfast. One hour and 3 h following the intake of Levodopa, a set of mfBIA measurements was again recorded.

#### 2.4.2 Healthy controls

One set of mf‐BIA measurements was taken when the subjects arrived and following the UPDRS assessment.

### 2.5 Data handling

Analysis of the mf‐BIA data was carried out using the ImpediMed Inc software (Impedimed SFB7). First, the Cole‐Cole plot was analyzed for normal distribution, and the resistance (*R*)/reactance (Xc) plots were examined. Following this, the Centre Frequency (fc) and the extracellular resistance (Re) were determined from the Cole‐Cole plot, and intracellular resistance (Ri) was calculated from the formula: Ri = (Re × *R*∞/Re–*R*∞). Membrane capacitance (M_c_) was, furthermore, calculated from the following formula: fc = 1/(2π x Mc x (Re + Ri)). A detailed analysis was performed at 50 kHz with measurement of *R* and Xc, and calculation of impedance (*Z*): *Z* = Square Root (R^2^ + Xc^2^). Finally, the phase angle (PA) was calculated: PA = arctan (Xc/*R*) with units in degrees. The mf‐BIA parameters were interpreted in terms of muscle mass, hydration, contraction level, membrane activity, and metabolic activity.

#### 2.5.1 Correlations between patient and healthy control subject groups

Using GraphPad Prism 9.2, the data was handled using both an ANOVA and regression analyses (Pearson's correlation rank correlation coefficient), as well as linear regression and the regression coefficient from this.

#### 2.5.2 Self‐assessment via questionnaires

PainDetect was only used to make certain that pain would not affect muscle use and, therefore, only used in the selection process of subjects.

## 3 RESULTS

### 3.1 Subjects

The 20 patients consisted of 13 men and 7 women. The mean age with SD was 71 ± 7 years, range 60–84 years, for the men, and 65 ± 7 years, range 51–73 years, for the women. The control group consisted of 13 men with a mean age and SD 71 ± 7 years, range of 60–84 years, and 7 women with a mean age and SD of 65 ± 7 years, range of 51–73 years.

When looking at the data for the healthy controls and earlier published data on a healthy population (Bartels et al., 2015) the data were found to be similar, taking into account this smaller sample size and a slightly older subject group.

This study was designed based on power calculations on mfBIA data which predicted the sample size in this study should be adequate. A statistical analysis of the mfBIA parameters for the healthy controls (HC) as well as the PD subjects at times 0, 1, and 3, revealed a number of interesting findings, particularly with regards to the R_i_, R_e_, and Mc parameters (see Tables 1, 2, 3, 4, 5, 6). The mfBIA data can be found in the appendix (see Appendix A).

**TABLE 1:** Statistically significant mfBIA parameters given by *p*‐values for male healthy controls (HC) versus male Parkinson's subjects (PD) of m. biceps at three different time points; PD0 = defined as pragmatic off = free of medicine for 3 × T_1/2_ (12 h), and both PD1 at 1 and PD3 at 3 h following medicine intake

Males	HC	PD0	PD1	PD3
HC	NS	R_i_ *p* = 0.0003; R_e_ *p* = 0.0001; *Z p* = 0.0001; *R p* = 0.0001; *fc p* = 0.0001; Mc *p* = 0.009	R_i_ *p* = 0.005; R_e_ *p* = 0.0006; *Z p* = 0.001; *R p* = 0.001; Mc *p* = 0.015	R_i_ *p* = 0.0007; R_e_ *p* = 0.0001; *Z p* = 0.0002; *R p* = 0.0002; Mc *p* = 0.017
PD0	—	NS	NS	NS
PD1	—	—	NS	NS
PD3	—	—	—	NS

**TABLE 2:** Statistically significant mfBIA parameters given by *p*‐values for female healthy controls (HC) versus female Parkinson's subjects (PD) of m. biceps at three different time points; PD0 = defined as pragmatic off = free of medicine for 3 × T_1/2_ (12 h), and both PD1 at 1 and PD3 at 3 h following medicine intake

Females	HC	PD0	PD1	PD3
HC	NS	R_i_ *p* = 0.0001; R_e_ *p* = 0.001; *Z p* = 0.002; *R p* = 0.002; Mc *p* = 0.005	R_i_ *p* = 0.0001; R_e_ *p* = 0.001; *Z p* = 0.002; *R p* = 0.002; Mc *p* = 0.0031	R_i_ *p* = 0.0001; R_e_ *p* = 0.002; *Z p* = 0.002; *R p* = 0.002; Mc *p* = 0.0069
PD0	—	NS	NS	NS
PD1	—	—	NS	NS
PD3	—	—	—	NS

**TABLE 3:** Statistically significant mfBIA parameters given by *p*‐values for male healthy controls (HC) versus male Parkinson's subjects (PD) of m. triceps at three different time points; PD0 = defined as pragmatic off = free of medicine for 3 × T_1/2_ (12 h), and both PD1 at 1 and PD3 at 3 h following medicine intake

Males	HC	PD0	PD1	PD3
HC	NS	R_i_ *p* = 0.02; R_e_ *p* = 0.02; *Z p* = 0.01; *R p* = 0.01; Mc *p* = 0.04	R_e_ *p* = 0.03; *Z p* = 0.02; *R p* = 0.02; Mc *p* = 0.01	R_i_ *p* = 0.01; *Z p* = 0.03; *R p* = 0.02; Mc *p* = 0.03
PD0	—	NS	NS	NS
PD1	—	—	NS	NS
PD3	—	—	—	NS

**TABLE 4:** Statistically significant mfBIA parameters given by *p*‐values for female healthy controls (HC) versus female Parkinson's subjects (PD) of m. triceps at three different time points; PD0 = defined as pragmatic off = free of medicine for 3 × T_1/2_ (12 h), and both PD1 at 1 and PD3 at 3 h following medicine intake

Females	HC	PD0	PD1	PD3
HC	NS	NS	NS	NS
PD0	—	NS	NS	NS
PD1	—	—	NS	NS
PD3	—	—	—	NS

**TABLE 5:** Statistically significant mfBIA parameters given by *p*‐values for male healthy controls (HC) versus male Parkinson's subjects (PD) of m. extensor carpi radialis longus at three different time points; PD0 = defined as pragmatic off = free of medicine for 3 × T_1/2_ (12 h), and both PD1 at 1 and PD3 at 3 h following medicine intake

Males	HC	PD0	PD1	PD3
HC	NS	NS	NS	NS
PD0	—	NS	NS	NS
PD1	—	—	NS	NS
PD3	—	—	—	NS

**TABLE 6:** Statistically significant mfBIA parameters given by *p*‐values for female healthy controls (HC) versus female Parkinson's subjects (PD) of m. extensor carpi radialis longus at three different time points; PD0 = defined as pragmatic off = free of medicine for 3 × T_1/2_ (12 h), and both PD1 at 1 and PD3 at 3 h following medicine intake

Females	HC	PD0	PD1	PD3
HC	NS	NS	NS	NS
PD0	—	NS	NS	NS
PD1	—	—	NS	NS
PD3	—	—	—	NS

### 3.2 m.Biceps

The mfBIA statistical analysis and level of significance for m.biceps for the male and female subjects at the given time points can be seen below in Tables 1 and 2.

### 3.3 m.Triceps

The mfBIA statistical analysis and level of significance for m.triceps for the male and female subjects at the given time points can be seen below in Tables 3 and 4. It should be noted for this muscle that recordings were successfully made for 12 of the 13 male PD subjects.

### 3.4 m.Extensor carpi radialis longus

The mfBIA statistical analysis and level of significance for m.extensor carpi radialis longus for the male and female subjects at the given time points can be seen below in Tables 5 and 6.

(Tables 5 and 6 near here)

UPDRS scores are designed to monitor impairment with PD. It is not directly related to the condition of a single muscle and for this reason, we do not find it relevant to correlate the UPDRS score for these PD subjects with our mfBIA parameters.

## 4 DISCUSSION

This study is the first to assess muscle health using mfBIA on PD subjects off and medication. It shows that there are muscle‐specific changes with medication, and highlights a number of mfBIA parameters that are affected in only m.biceps brachii.

It is interesting to note that the significant effects of medication on mfBIA parameters were found for the flexor muscle (m. biceps), whilst both upper limb extensors in this study showed no significant change. However, to the best of our knowledge, there is no prior specific study in PD patients focusing on disease effects on muscle power, i.e. flexors versus extensors and upper versus lower limbs. One might speculate that PD has an effect on the flexors in the upper limbs and the extensors in the lower limbs, mirroring the distribution of muscle power effects and spasticity in patients with pyramidal tract dysfunction (Li et al., 2018; Underwood & Parr‐Brownlie, 2021). Further studies in this field are clearly now needed. What is known is that the innervation for m.biceps is by the musculocutaneous nerve (C 5–6), whilst the innervation for both m.triceps and m.extensor carpi radialis longus is by the radial nerve (C 5–8), which is a branch of the brachial plexus. However, if this difference has any significance remains to be established.

An earlier study by this group has already demonstrated, in a larger healthy adult population spanning 20–69 years in both males and females, that mfBIA parameters differ with gender (Bartels et al., 2019). For all the muscles measured in this study, we found, as expected, the gender difference for mfBIA parameters. Likewise, this technique is not only non‐invasive, but it is also fast and has been shown over the years to be reliable and repeatable, and can be recommended for use in the clinic (Bartels et al., 2015; Bartels et al., 2019; Harrison et al., 2015; Nescolarde et al., 2013; Nescolarde et al., 2015).

The first hypothesis proposed by this study stated that cellular energy metabolism would be higher in PD subjects compared with healthy controls and that would be shown by an elevated Ri and a lower Mc parameter. It is clear that PD subjects do indeed exhibit an elevated Ri and a lower Mc, confirming this hypothesis, however, this was only found to be true for m.biceps.

The second hypothesis predicted that increased muscle rigidity in PD subjects would affect both the fc and Re parameters compared with healthy controls, resulting in an increase in their value. In this instance, only a significant difference between fc for PD subjects at Time 0 and that of healthy Controls was found (m.biceps), and this showed a decrease rather than an increase. However, the Re parameter showed a significant increase in both men and women for PD subjects compared with healthy controls (m.biceps), a change that was not only expected when subjects were off their medication but one that was maintained even though their medication was re‐introduced.

What does this finding mean for PD subjects? The following text refers solely to the m.biceps flexor muscle, where significant changes were observed.

Prior to taking the PD drug livodopa, where the level of dopamine in the basal ganglia is lower than normal, fc is seen to be higher in the PD men compared to the HC group. Following medication, this difference disappears, which is in accordance with the change in muscle rigidity one might expect for these subjects. There is a tendency in women toward this, but the low number of women in this study may prevent this difference from being significant.

Ri in PD stays higher and unchanged compared to the HC group in the unmedicated and medicated states. This indicates that PD muscle is metabolically more active than HC muscle since Ri is directly related to VO2max, a definition of maximum oxygen consumption by individuals (amount of oxygen consumed per kilo of body weight every minute) (Stahn et al., 2008).

The finding of a higher Ri is also linked with the change in membrane capacitance (Mc). A lower Mc in PD in all states compared to the HC group has been shown in this study. Mc relates directly to membrane transport, and a lower Mc indicates more activity over the membrane. Since Ri is high this speaks against cellular death processes and more toward metabolic activity and nutrient uptake across cellular membranes. The consequence of all of this is an osmotic gradient with fluid moving into cells during nutrient transport (Howell et al., 1993; Whitehead et al., 1998; Zierler et al., 1985).

Re is also higher in PD subjects compared to the HC group in off‐medicine and all medication states. This indicates that there is either impaired diffusion in the interstitial compartment or less hydration (Tonkovic et al., 2000). The latter could be created by high membrane activity.

## 5 CONCLUSION

The important finding in this study is that the upper limb flexor muscle in PD shows a difference when compared to HC and the extensor muscles. The method looks promising in the assessment of PD patients since it is objective, non‐invasive, reliable, and easy to use. These findings now need to be fully understood from a neurological perspective.

## AUTHOR CONTRIBUTIONS

MC, EB, AH, AML and HS contributed to the conception and design of the work. MC and EB performed the measurements. MC, EB, and AH, analyzed the data. MC drafted the initial manuscript. All authors have read, revised, and approved the manuscript.

## FUNDING INFORMATION

The Parker Institute was supported by core funding from the Oak Foundation (Ocay‐13‐309). The project was supported by Erna Hamiltons Fond.

## CONFLICT OF INTEREST

None of the authors had any conflicts of interest in this study.

## ETHICS STATEMENT

The study followed the guidelines set by the Helsinki Declaration 2013 (https://www.wma.net/policies‐post/wma‐declaration‐of‐helsinki‐ethical‐principles‐for‐medical‐research‐involving‐human‐subjects/) and all subjects gave informed written consent prior to participating in the study. The study was approved by the Capital Region of Denmark's Ethics Committee (No. H‐17021637) and was registered at the Danish Data Protection Agency. All data was collected and handled according to The Act on Processing of Personal Data (Act No. 429 of 31 May 2000 with amendments [latest 2018]).

## APPENDIX A

### A .1 M Biceps Brachii

The population of males and females were found to be normally distributed.

### A.2 PD data

**Table:**

	*Z*	*R*	Fc	PA	Ri	Re	Xc	Mc
Men								
Time 0	91.1 ± 31.1	90.5 ± 31.3	51.1 ± 9.6	6.3 ± 3.8	313.6 ± 187.8	105.3 ± 33.4	8.8 ± 2.9	10.5 ± 7.6
Time 1	88.4 ± 36.4	87.9 ± 36.6	58.7 ± 36.8	6.6 ± 3.6	291.9 ± 219.5	103.1 ± 38.6	8.9 ± 2.9	11.5 ± 7.4
Time 3	80.9 ± 27.8	80.2 ± 27.9	54.2 ± 29.6	7.3 ± 3.6	235.2 ± 146.0	95.7 ± 30.9	9.4 ± 3.0	12.4 ± 7.1
Women								
Time 0	128.5 ± 42.0	128.2 ± 42.1	71.9 ± 27.8	3.9 ± 2.1	638.0 ± 415.7	140.6 ± 42.0	7.8 ± 2.1	4.5 ± 3.4
Time 1	123.1 ± 37.6	122.8 ± 37.8	64.7 ± 10.7	4.1 ± 1.6	503.5 ± 276.1	136.4 ± 38.8	8.0 ± 1.1	4.9 ± 2.7
Time 3	131.3 ± 43.9	131.0 ± 44.0	59.8 ± 25.3	3.9 ± 2.2	597.8 ± 342.0	145.8 ± 46.6	7.8 ± 2.4	5.2 ± 3.1

### A.3 HC data

**Table:**

	*Z*	*R*	Fc	PA	Ri	Re	Xc	Mc
Men	39.0 ± 4.6	38.4 ± 4.5	87.1 ± 19.3	9.8 ± 1.3	55.7 ± 7.5	45.9 ± 6.3	6.7 ± 1.4	18.9 ± 4.6
Women	44.9 ± 3.4	44.3 ± 3.3	90.3 ± 16.9	9.6 ± 2.0	69.5 ± 14.3	52.2 ± 4.6	7.5 ± 1.7	15.3 ± 4.4

### A.4 M Triceps

The population of males and females was found to be normally distributed. One of the PD subjects had missing data for this muscle.

### A.5 PD data

**Table:**

	*Z*	*R*	Fc	PA	Ri	Re	Xc	Mc
Men								
Time 0	95.8 ± 34.6	95.4 ± 34.8	50.1 ± 18.6	5.0 ± 2.8	406.4 ± 255.1	108.2 ± 36.6	7.2 ± 1.9	9.4 ± 6.3
Time 1	97.0 ± 38.4	96.7 ± 38.6	57.4 ± 15.6	5.2 ± 2.6	467.6 ± 468.1	108.5 ± 38.8	7.5 ± 2.4	8.1 ± 5.0
Time 3	91.8 ± 34.1	91.4 ± 34.3	52.0 ± 18.4	4.9 ± 3.4	451.5 ± 299.0	102.6 ± 35.8	6.6 ± 2.0	9.0 ± 7.0
Women								
Time 0	119.0 ± 23.9	118.7 ± 23.8	64.7 ± 21.3	3.6 ± 2.1	547.6 ± 228.0	132.4 ± 25.9	7.5 ± 4.3	4.8 ± 3.0
Time 1	125.1 ± 38.6	124.8 ± 38.7	80.6 ± 29.5	4.0 ± 2.5	623.6 ± 429.3	137.4 ± 38.6	7.8 ± 3.7	4.5 ± 4.2
Time 3	127.3 ± 48.9	127.1 ± 49.0	70.2 ± 27.3	2.9 ± 1.8	785.6 ± 484.1	137.2 ± 49.9	5.7 ± 2.1	4.0 ± 3.

### A.6 HC data

**Table:**

	*Z*	*R*	Fc	PA	Ri	Re	Xc	Mc
Men	66.9 ± 15.7	66.4 ± 15.8	45.7 ± 15.2	7.1 ± 2.4	201.8 ± 113.4	79.5 ± 16.9	7.9 ± 2.4	16.1 ± 8.7
Women	104.7 ± 17.3	104.5 ± 17.3	78.6 ± 27.0	3.5 ± 0.7	446.0 ± 111.7	114.6 ± 17.8	6.3 ± 0.9	4.3 ± 2.1

### A.7 M extensor carpi radialis longus

The population of males and females was found to be normally distributed.

### A.8 PD data

**Table:**

	*Z*	*R*	Fc	PA	Ri	Re	Xc	Mc
Men								
Time 0	56.2 ± 16.8	55.4 ± 16.9	53.0 ± 8.8	9.8 ± 3.0	113.0 ± 75.1	69.0 ± 19.2	9.2 ± 2.7	19.5 ± 6.8
Time 1	48.3 ± 11.8	47.5 ± 12.0	59.7 ± 12.6	10.6 ± 3.0	85.1 ± 47.5	59.5 ± 13.8	8.5 ± 1.9	21.2 ± 6.7
Time 3	50.7 ± 11.0	49.9 ± 11.0	49.6 ± 7.4	10.5 ± 2.5	88.8 ± 38.6	63.8 ± 13.2	9.1 ± 2.1	23.1 ± 6.5
Women								
Time 0	77.1 ± 12.0	76.5 ± 12.3	73.2 ± 21.5	6.8 ± 3.3	224.1 ± 138.2	88.9 ± 9.5	8.6 ± 3.2	9.3 ± 5.1
Time 1	72.7 ± 9.2	72.1 ± 9.4	64.6 ± 15.1	6.9 ± 3.6	217.6 ± 140.0	84.7 ± 10.0	8.4 ± 4.2	10.2 ± 4.7
Time 3	72.3 ± 11.3	63.9 ± 27.2	69.3 ± 35.0	7.4 ± 3.4	190.0 ± 118.6	85.6 ± 11.7	8.9 ± 3.8	11.6 ± 6.3

### A.9 HC data

**Table:**

	*Z*	*R*	Fc	PA	Ri	Re	Xc	Mc
Men	44.8 ± 11.5	43.8 ± 11.5	58.9 ± 16.7	11.9 ± 2.9	68.7 ± 45.5	56.7 ± 15.1	8.9 ± 2.6	25.9 ± 9.9
Women	67.1 ± 17.0	66.6 ± 17.0	63.8 ± 21.1	6.6 ± 1.6	167.8 ± 80.5	78.0 ± 18.6	7.5 ± 1.6	13.0 ± 7.9
